# Chelator-Based Parameterization of the 12-6-4 Lennard-Jones Molecular Mechanics Potential for More Realistic Metal Ion–Protein Interactions

**DOI:** 10.1021/acs.jctc.1c00898

**Published:** 2022-03-23

**Authors:** Paulius Kantakevičius, Calvin Mathiah, Linus O. Johannissen, Sam Hay

**Affiliations:** Manchester Institute of Biotechnology and Department of Chemistry, The University of Manchester, Manchester M13 9PL, U.K.

## Abstract

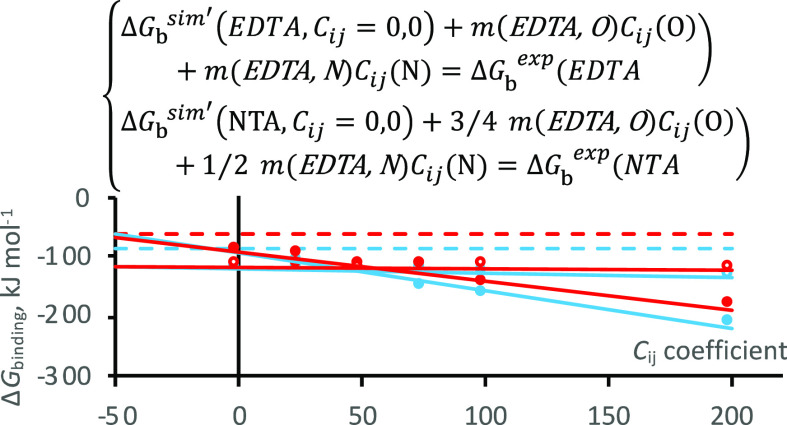

Metal ions are associated with a variety of proteins and play critical roles in a wide range of biochemical processes. There are multiple ways to study and quantify protein–metal ion interactions, including molecular dynamics simulations. Recently, the AMBER molecular mechanics forcefield was modified to include a 12-6-4 Lennard-Jones potential, which allows for a better description of nonbonded terms through the additional pairwise *C_ij_* coefficients. Here, we demonstrate a method of generating *C_ij_* parameters that allows parametrization of specific metal ion-ligating groups in order to tune binding energies computed by thermodynamic integration. The new *C_ij_* coefficients were tested on a series of chelators: ethylenediaminetetraacetic acid, nitrilotriacetic acid, egtazic acid, and the EF1 loop peptides from the proteins lanmodulin and calmodulin. The new parameters show significant improvements in computed binding energies relative to existing force fields and produce coordination numbers and ion-oxygen distances that are in good agreement with experimental values. This parametrization method should be extensible to a range of other systems and could be readily adapted to tune properties other than binding energies.

## Introduction

Metal ions are thought to be associated with around 50% of all proteins.^1^ They play important roles in a multitude of biologically significant processes, such as enzyme catalysis and signal transduction.^2−4^ Inclusion of metal ions into molecular mechanics (MM) force fields used for molecular dynamics (MD) simulations poses specific challenges because of the high polarizability of metal ions, which can take part in charge transfers, coordination number (CN) changes, and ligand swapping.^5−7^ Several approaches have been developed for simulating metal ions, but these typically lack transferability between systems or simulate one property accurately at the expense of others. For example, explicitly bonded models treat metal–ligand coordination as immutable bonds, which does not allow for ligand and CN changes.^8^ Such methods therefore cannot provide insight into coordination dynamics, and multiple simulations with different bonding parameters are required to determine optimal CN in a given system.^7^ Another commonly used approach is to rely on nonbonded terms that treat metal–ligand coordination as nonbonded interactions facilitated by Lennard-Jones (LJ) and Coulombic terms. The 12-6LJ nonbonded model is the default in most MM force fields and defines the standard LJ potential using two pairwise parameters for each type of atom–atom interaction (eq 1). This approach allows for the metal ions to switch both CN and ligands but lacks charge transfer effects and polarizability. Nonetheless, this approach is commonly used for MD simulations as it is computationally efficient and does not require assumptions about the ligands coordinated to the metal ion, which usually require previous knowledge about the simulated system.

Recently, an array of new 12-6LJ parameters were developed to describe divalent metal ions. The “12-6LJ HFE” parameters accurately reproduced hydration free energies (HFEs), the “12-6LJ IOD” parameters reproduced ion-oxygen distances (IODs), while the “12-6LJ CM” parameters were designed as a compromise between both.^6^ As the 12-6LJ potential does not accurately reproduce both HFE and IOD experimental values with a single set of parameters, this led to the development of the new 12-6-4LJ potential.^6,9^ This contains an additional pairwise parameter, which accounts for the charge induced dipole and dipole induced dipoles that are neglected in the 12-6LJ potential (eq 2, below). This addition to the force field made it possible to use a single set of parameters to adequately describe HFE, IOD, and CN values in three commonly used water models, TIP3P, SPC/E, and TIP4Pew.^9−11^ However, the *C_ij_* coefficients of the new *C_ij_*/*r_ij_*^4^ term were parameterized only for the interactions with the water oxygen atoms, whereas parameters for other types of atoms were adopted from molecular polarizability tensor calculations.^12^ In a subsequent study of metal ion–nucleotide interactions, it was shown that the adopted *C_ij_* coefficients may not produce accurate binding energies as they overestimated the binding energies to adenine, guanine, and phosphate by up to 20 kJ mol^–1^.^13^ These authors were able to generate a new set of *C_ij_* coefficients that increased the accuracy of estimated binding energies (±0.5 kJ mol^–1^ relative to experimental values) to nucleotides and phosphate without affecting the metal–water interactions, as the metal ion parameters contain separate *C_ij_* coefficients for each atom type. This suggests that further parameterization of these *C_ij_* coefficients with amino acids offers a reasonable approach to improve the accuracy of MD simulations of metal ions.

**Figure 1:**
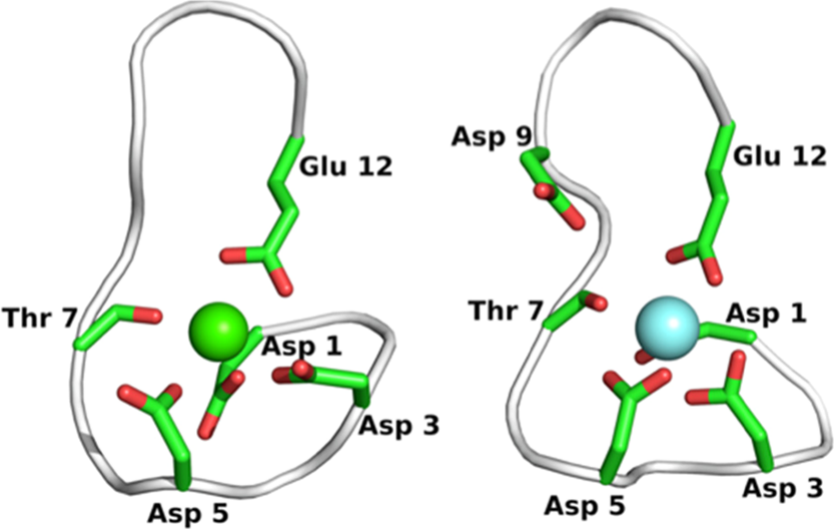
Metal ion coordination in the EF1 loops of CaM (left) and LanM (right). Ca^2+^ is bound to CaM (PDB 1CLL) and Y^3+^ is bound to LanM (NMR conformer 1 of PDB 6MI5).^15,16^

**Figure 2:**
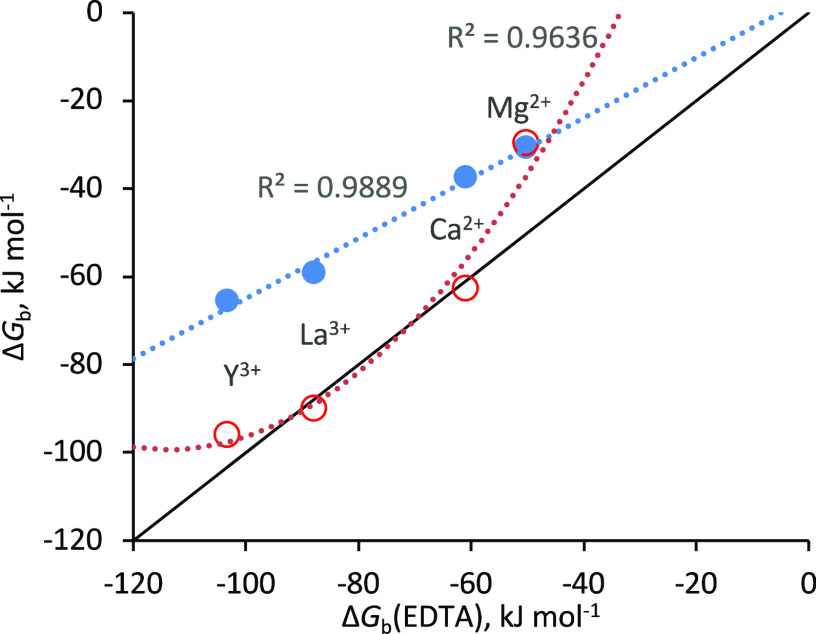
Experimental binding energies for metal binding to NTA (blue) and EGTA (red) plotted against those for EDTA. Black line shows the diagonal (*x* = *y*). NTA data are fitted to a linear function with a slope of 0.68 and a *y* intercept of +3 kJ mol^–1^ and the EGTA data are fitted to a second-order polynomial to guide the eye. Binding affinities are given in Table S1.

In this study we have developed a new set of pairwise *C_ij_* coefficients for Ca^2+^, Mg^2+^, Y^3+^, and La^3+^ ions using the 12-6-4LJ nonbonded potential. These metals were chosen to investigate metal binding to a recently discovered lanthanide selective protein, lanmodulin (LanM), which is homologous to the calcium binding protein calmodulin (CaM).^14,15^ Ethylenediaminetetraacetic acid (EDTA) and nitrilotriacetic acid (NTA) were used to determine the parameters, as these contain metal ion ligating carboxylate groups similar to those found in glutamate and aspartate (Figure 1 and Figure S1) and accurate binding affinities are available (Figure 2, Table S1). A linear equation system based on the ratios of ligating oxygens and tertiary nitrogen atoms was developed to deconvolute the contribution from each ligating atom type. The new *C_ij_* coefficients were tested against another chelator, egtazic acid (EGTA), and the EF1 loop peptides of LanM and CaM. They give binding energies that are significantly closer to experimental values than the 12-6LJ CM/IOD parameter sets or the 12-6-4LJ potential with default *C_ij_* coefficients and reproduce the observed metal ion selectivity in LanM.

## Computational Methods

### MD Simulations and Thermodynamic Integration

The nonbonded potentials used by the AMBER ff14SB force field are described by eqs 1 and 2, which contain 12-6 and 12-6-4LJ terms, respectively:^17,18^
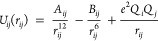
1
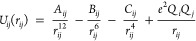
2Essentially, *A_ij_*/*r_ij_*^12^ is a repulsive term that prevents the attraction from becoming too strong at short distances, *B_ij_*/*r_ij_*^6^ is an attractive term derived from London dispersion forces, *C_ij_*/*r_ij_*^4^ accounts for ion-induced dipoles and the *e*^2^*Q_i_Q_j_*/*r_ij_* Coulombic term accounts for the electrostatic interactions between the atoms.^9,19^

Thermodynamic integration (TI) was used to calculate Ca^2+^, Mg^2+^, Y^3+^, and La^3+^ ion HFEs and binding energies, according to the standard thermodynamic cycle shown in Figure S2. All simulations were carried out using AMBER18 with the ff14SB force field and TIP3P solvation extended at least 15 Å from the solute.^17,18,20^ Chelator parameters were determined using the Antechamber package from AmberTools19^17^ with charges calculated using the AM1-BCC charge model.^21^ Charges are given in Table S2. Particle Mesh Ewald was used for long-range nonbonded electrostatic interactions with a cut off distance of 10 Å.^22^ The SHAKE algorithm was used to constraint all covalent bonds that involve hydrogen atoms and a 2 fs timestep was used.^23^ The temperature was maintained at 300 K using the Langevin thermostat with a 2.0 ps^–1^ collision frequency and the pressure at 1 atm using the Berendsen barostat.^17,24^ After initial TI testing it was observed that fewer intermediate λ states were required to reach a converged Δ*G*_VdW_ value than for the Δ*G*_ele+pol_ term, so 9 λ steps were used to calculate Δ*G*_VdW_ and 12 for Δ*G*_ele+pol_ with Gaussian integration.^17^ The simulations to determine Δ*G*_ele+pol_ were run in triplicate to ensure sufficient sampling and to allow error estimation. The lengths of EM, *NVT*, *NPT* and MD sampling per intermediate λ state are outlined in Table S3. More details on the simulation parameters and protocols are available in the Supporting Information.

We tested three existing parameter sets: 12-6LJ HFE, 12-6LJ CM/IOD, and the 12-6-4LJ standard parameters. The 12-6LJ HFE parameters should provide accurate ion HFEs but are known to produce IODs that are shorter than experimental values by an average of 0.27 and 0.29 Å for divalent and trivalent ions, respectively.^10^ The 12-6LJ CM parameters should provide a compromise between IOD and HFE, but these are not available for trivalent ions. The 12-6-4LJ parameters were reported to reproduce accurate IODs and HFEs.^9^ Even though 12-6LJ parameters are expected to produce poorer results, they were included as a benchmark.

In order to calculate average IODs and CNs, data were taken from the TI simulations with λ = 0.00922, which reproduced the known IOD and CN of fully charged Ca^2+^ and Mg^2+^ ions in water (Table S4). For full-length LanM, a separate 10 ns MD simulation was used. IODs and CNs were calculated from radial distribution functions (RDFs), which were calculated to a resolution of 0.01 Å using VMD.^25,26^ The IODs were taken as the peak of a quadratic fit applied to ±0.1 Å of the first peak of the RDF, and CN (taken as the number of atoms within the first coordination sphere around the metal ion) was calculated by integrating the RDF from 0 to the first minimum.

### Experimental Benchmarking

The 12-6LJ and 12-6-4LJ parameters were obtained using experimental HFE values, so we used the same values for our comparisons.^6,9,10,27^ EDTA and NTA metal ion stability constants (*K*_1_) were taken from NIST reports.^28,29^ For EGTA, no NIST values were found, so values from the Dojindo metal chelate affinity report were taken.^30^ Dissociation constants (*K*_d_) for LanM and CaM were taken from refs (14,31−33). Note that these will give average binding energies for the four EF loops in each protein. All experimental values were converted to binding energies in kJ mol^–1^ at 298 K (Table S1) assuming:
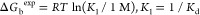
3

## Results and Discussion

**Figure 3:**
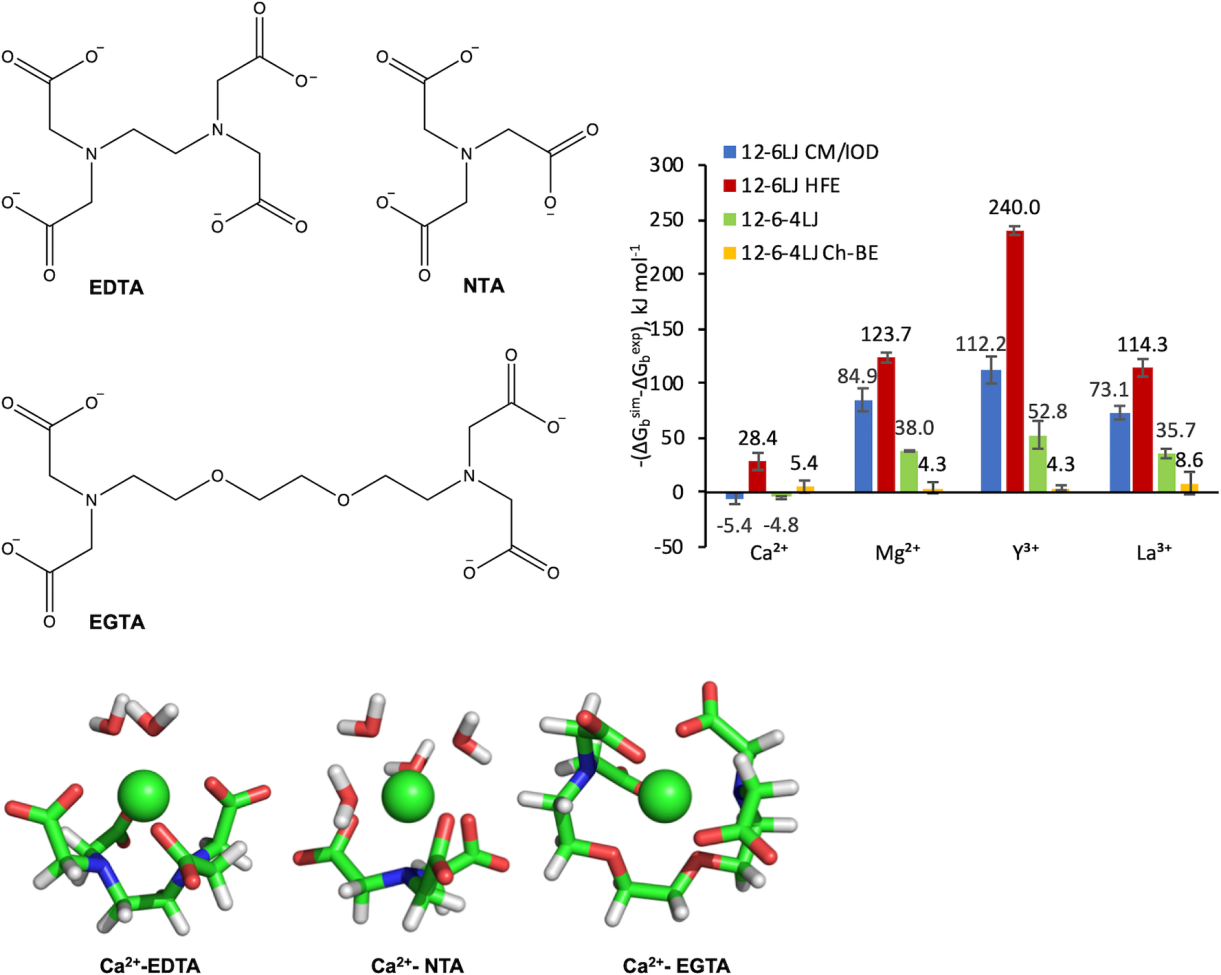
*Left*, the structures of EDTA, NTA, and EGTA and *below*, snapshots taken from MD simulations of the Ca^2+^ bound chelators performed with 12-6-4LJ default *C_ij_* coefficients. *Right*, the difference between experimental and computed EDTA-metal ion binding energies obtained using four sets of parameters. 12-6LJ CM was used for Ca^2+^ and Mg^2+^ while 12-6LJ IOD was used for Y^3+^ and La^3+^. “12-6-4LJ Ch-BE” denotes the parameters generated during this study.

Initially, EDTA binding energies for Ca^2+^, Mg^2+^, Y^3+^ and La^3+^ were computed by TI using three established parameter sets:^6,9,10^ 12-6LJ HFE and 12-6-4LJ for all metals and 12-6LJ CM for Ca^2+^, Mg^2+^, or 12-6LJ IOD for Y^3+^ and La^3+^ (12-6LJ CM parameters not available for trivalent ions). The difference between the computed EDTA-metal ion binding energies (Δ*G*_b_^sim^) and the experimental values in Figure 2 (Δ*G*_b_^exp^) is shown in Figure 3. HFEs are given in Table S5 and absolute binding energies are given in Table S6. In most cases, all three parameter sets significantly overestimate the binding energy (producing more negative values), although they produced relatively accurate binding energies for Ca^2+^. While 12-6-4LJ produced the best results, there is significant room available for improvement as this showed errors ranging from +4.8 to −52.8 kJ mol^–1^.

To increase the accuracy of the TI calculations for the binding energy between chelators and metal ions, we chose to re-parameterize specific *C_ij_* coefficients of the 12-6-4LJ parameter set (eq 2) as this allows the modification of the pairwise interactions between the metal ion and specific atom types (i.e., ligating atoms) without affecting other interatomic interactions and the HFE.^13^ EDTA, NTA, and EGTA can coordinate metal ions using both carboxylate groups and tertiary nitrogen atoms (Figure 3). As the ligating groups are in similar chemical environments in the three chelators, we reasoned that a single set of ligating oxygen and nitrogen parameters can be shared between these molecules. EGTA also contains ether groups, which we did not specifically parameterize in this study. *C_ij_* coefficients for the carboxylate oxygen and the tertiary nitrogen were parameterized using TI simulations of EDTA and NTA using the 12-6-4LJ standard parameters. EGTA was then used for benchmarking.

**Figure 4:**
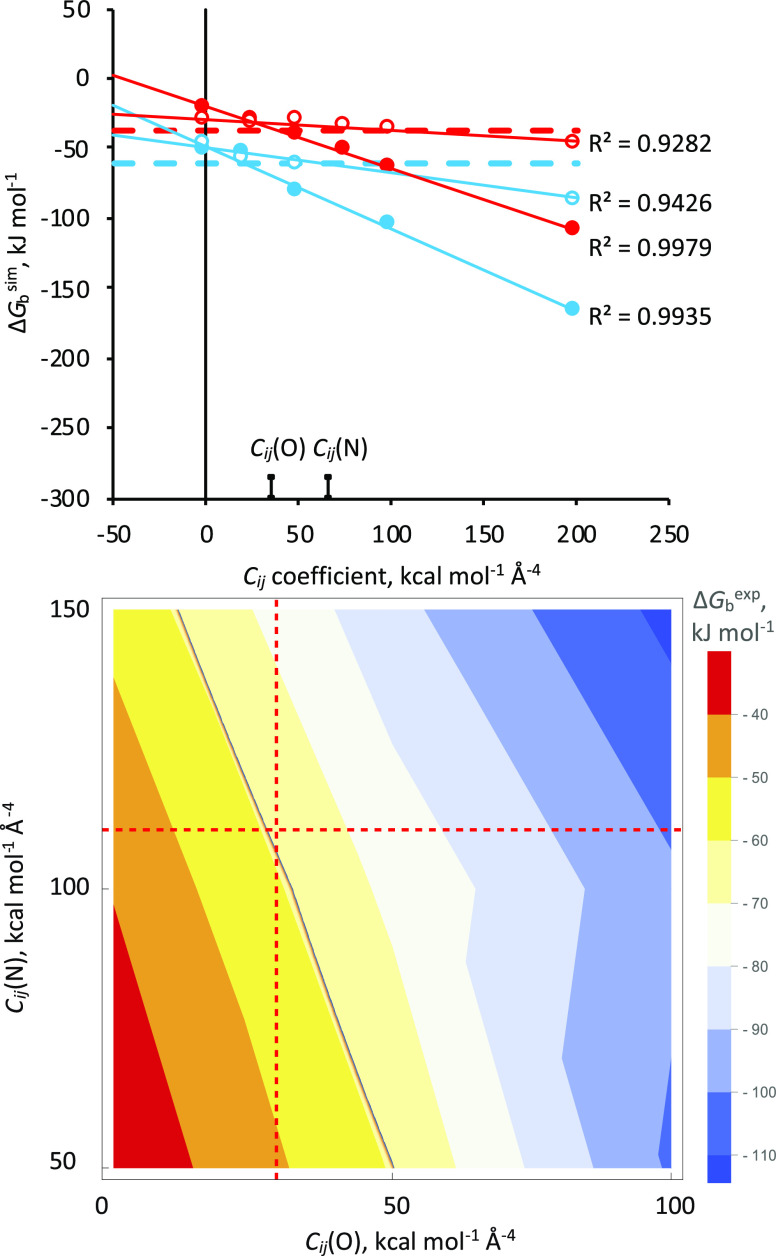
*Top*, Computed binding energies for the chelation of Ca^2+^ by EDTA (blue) and NTA (red) as a function of the ligating oxygen and nitrogen 12-6-4LJ *C_ij_* values. Filled symbols are for oxygen and open symbols for nitrogen. Solid lines are linear fits to the data and the horizontal dashed lines are the experimental Δ*G*_b_^exp^ values from Table 1. Default *C_ij_* values are labeled. *Bottom*, 2D plot of Δ*G*_b_^sim^ for the chelation of Ca^2+^ by EDTA versus the ligating oxygen and nitrogen 12-6-4LJ *C_ij_* values. Contour corresponding to the Δ*G*_b_^exp^ value is shown in black and the new Ch-BE values from Table 2 are shown as red dashed lines.

Figure 4 (top panel) shows Δ*G*_b_^sim^ values for Ca^2+^ coordination by EDTA and NTA, which were computed at different ligating oxygen and nitrogen *C_ij_* values while holding all other *C_ij_* parameters to their default 12–6-4-LJ values, that is, *C_ij_*(O) was set to default when varying *C*_ij_(N) and vice versa. Data for other metal ions are shown in Figures S4–S6. These data all show linear dependences of Δ*G*_b_^sim^ on *C_ij_*(O), so they were fitted to a linear function, with the gradients, *m* given in Table 1. While there is not a strong dependence of Δ*G*_b_^sim^ on *C_ij_*(N) for some metals, these data were also fitted to a linear function for consistency. Binding free energies were also computed with *C_ij_* values for both ligating oxygen and nitrogen atoms set to 0 and these Δ*G*_b_^*sim*^(*C_ij_* = 0,0) values are also given in Table 1. In principle, the following relationship should then describe the experimental binding energy:
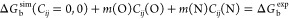
4

**Table 1:** Metal Binding Energies (kJ mol^–1^) for EDTA and NTA and Gradient (*m*) Values (kJ mol^–1^*C_ij_*^–1^) for the Data in Figures 4, S4–S6

	Δ*G*_b_^exp^^28,29^		Δ*G*_b_^sim^(*C*_*ij* default_), Δ*G*_b_^sim^(*C_ij_* = 0,0)^a^		*m*(O)		*m*(N)	
metal	EDTA	NTA	EDTA	NTA	EDTA	NTA	EDTA	NTA
Ca^2+^	–60.8	–37.5	–56.0, −41.3 (−24.0)	–32.7, −17.6	–0.593	–0.442	–0.177	–0.081
Mg^2+^	–50.1	–30.6	–88.1, −37.6 (−21.8)	–84.9, −52.5	–1.028	–0.842	–0.124	+0.077
Y^3+^	–103.2	–65.5	–156.0, −81.2 (−82.6)	–148.9, −107.1	–0.752	–0.691	–0.058	+0.008
La^3+^	–87.6	–59.1	–123.3, −85.0 (−79.0)	–115.4, −86.7	–0.632	–0.491	–0.056	–0.028

a Computed using standard parameters and with *C_ij_* for ligating oxygen and nitrogen groups both set to 0. The EDTA values in (parenthesis) are the Δ*G*_b_^sim^′(*C_ij_* = 0,0) values determined using eq 5.

A 2D plot of Δ*G*_b_^sim^ versus *C_ij_*(O) and *C*_ij_(N) for the chelation of Ca^2+^ by EDTA is shown in the bottom panel of Figure 4. The Δ*G*_b_^exp^ value corresponds to a contour in this plot, so to find a unique solution for *C_ij_*(O) and *C_ij_*(N)_,_eq 4 must be solved simultaneously for two or more different chelators with different binding energies and *m*(O) and *m*(N) values. However, when calculating the binding energies for EDTA and NTA with a range of *C_ij_* values, it became apparent that Δ*G*_b_^sim^(*C_ij_* = 0,0) values for NTA with Mg^2+^, Y^3+^ and La^3+^ were significantly larger than the experimental values (Table 1). Since larger *C_ij_* coefficients increase the binding energy, this meant that in order to satisfy eq 4 at least one of the *C_ij_* coefficients would have to become negative, which is not physically realistic. Further, some computed NTA binding energies are larger than the EDTA values for the same metal ion, in opposition to the experimental values (Table 1, Figure 2). This suggests that Δ*G*_b_^sim^(*C_ij_* = 0,0) are unreliable as computed. Instead, an equivalent binding free energy Δ*G*_b_^sim^′(*C_ij_* = 0,0) was determined by back-extrapolating from Δ*G*_b_^sim^(*C*_*ij* default_) determined using default 12-6-4-LJ values:
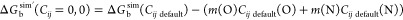
5

The EDTA Δ*G*_b_^*sim*^′(*C_ij_* = 0,0) values were determined using eq 5 and are given in Table 1. Equivalent values can be determined for NTA by scaling the EDTA values by the ratio of the experimental binding constants:
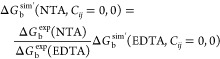
6

This approach ensures that the Δ*G*_b_^sim^′(*C_ij_* = 0,0) values for different chelators follow the experimental binding energy trends.

As seen in Table 1, for Mg^2+^ and Y^3+^, the NTA *m*(N) values are not physically realistic (non-negative). Visual inspection of the relevant MD trajectories suggested that these metal ions did not interact with the tertiary nitrogen atoms in the same way as EDTA (Figure S7), which likely leads to underestimation of these *m*(N) values.^34^ From the EDTA and NTA Ca^2+^ simulations, we noted that the ratios of the *m*(O) and *m*(N) values are approximately 3/4 and 1/2 for oxygen and nitrogen, respectively, thus mirroring the maximum CNs available (Figure 3).^35^ Based on this observation, we can modify eq 4 to scale the EDTA *m*(O) and *m*(N) values by 3/4 and 1/2, respectively, in order to describe the NTA binding energy. Making use of this approximation, and by substituting eqs 5 and 6 into eq 4, we can determine unique *C_ij_* values using only the EDTA TI simulation data and the experimental binding energies for EDTA and NTA in eq 7. The resulting ligating oxygen and nitrogen *C_ij_* coefficients are given in Table 2 and the Ca^2+^ values are plotted on the contour plot in Figure 4 to show they intersect at Δ*G*_b_^exp^. If different CNs are observed^36^ or suspected, eq 7 can be readily modified to use ratios other than 3/4 and 1/2. Examples of the analysis performed with alternative ratios is given in the Supporting Information.
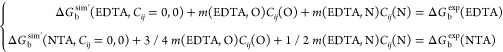
7

**Table 2:** Default and Newly Derived “Ch-BE” 12-6-4LJ Pairwise Oxygen and Nitrogen *C_ij_* Coefficients (kcal mol^–1^ Å^–4^) for Ca^2+^, Mg^2+^, Y^3+^, and La^3+^ Ligation by EDTA and NTA

metal	default		Ch-BE	
	O	N	O	N
Ca^2+^	34.4	65.9	29.2	110.6
Mg^2+^	52.4	100.3	12.3	126.1
Y^3+^	85.1	163.0	14.9	161.9
La^3+^	59.9	114.7	9.5	46.3

### Validation of New C_ij_ Coefficients

Our new *C_ij_* coefficients for the 12-6-4LJ nonbonded potential, which we denote “12-6-4LJ Ch-BE,” resulted in significant improvements to the TI-computed binding energies compared to the 12-6LJ CM/IOD, 12-6LJ HFE, and default 12-6-4LJ parameter sets. These are shown in Figure 3 and the absolute binding energy values are given in Table S6. Our new binding energies are all within 8.6 kJ mol^–1^ (∼2 kcal mol^–1^) of the experimental values, while the previously best results using the 12-6-4LJ default set differed by up to ∼50 kJ mol^–1^ from the experimental data. The average absolute error was reduced to 5.6 kJ mol^–1^ compared to 32.8 kJ mol^–1^ for the default 12-6-4LJ set.

**Figure 5:**
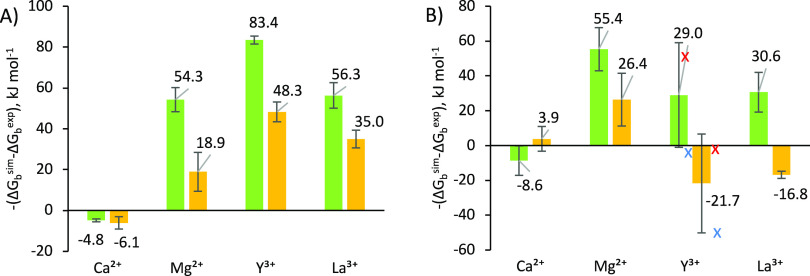
Difference between computed NTA (A) and EGTA (B) (Δ*G*_b_^sim^) and experimental (Δ*G*_b_^exp^) binding energies obtained using TI with default 12-6-4LJ (green) and 12-6-4-LJ Ch-BE (yellow) O and N *C_ij_* coefficients for the ligating oxygen and nitrogen groups. Energies are given below or above the bar and the red and blue crosses in (B) indicate the approximate energies for EGTA Y^3+^ in two different chelator conformations (Figure S8).

To ensure that the new *C_ij_* coefficients were not overfitted for EDTA, they were also tested on NTA (the NTA TI values were not used for parametrization; see eq 7) and a similar chelator EGTA (Figure 3). The difference in computed and experimental binding energies are shown in Figure 5 and the absolute binding energy values are given in Table S7. In each case, a clear improvement was observed. Overall, the “12-6-4LJ Ch-BE” parameter set reduced the absolute average binding energy errors from 49.7 to 27.1 kJ mol^–1^ for NTA and from 30.9 to 17.2 kJ mol^–1^ for EGTA, relative to the default 12-6-4LJ parameters. This represents ∼1.8-fold increase in accuracy for both NTA and EGTA, although it should be noted that there was significant variability in the EGTA values with Y^3+^ using both forcefields. This arose due to EGTA adopting another conformation in some simulations, which introduces additional hydrogen bonding between EGTA and solvent molecules. More detailed analysis of these results is discussed in the Supporting Information.

Next, we compared the 12-6-4LJ Ch-BE parameters to the 12-6-4LJ default and 12-6LJ CM/IOD parameter using TI simulations of metal ion binding to the CaM and LanM 12-residue EF1 loop peptides (Figures 1, S1). The 12-6LJ HFE set was not selected as it was the worst performing set of parameters with EDTA (Figure 3 and Table S6), and it is known to produce inaccurate IODs.^6,10^ Although 12-6LJ CM/IOD produced poorer results in EDTA-metal ion simulations than 12-6-4LJ (Figure 3) it was still included here as 12-6LJ potentials are more widely used. As the EF-hand peptides do not possess tertiary amines, only the new metal ion-oxygen pairwise *C_ij_* coefficients were used. These were used for the carboxylate oxygens, while the coordinating oxygen atom from the backbone of Thr 7 retained the default *C_ij_* coefficient as it is part of an amide bond which has not yet been re-parameterized. The length of the constant-pressure equilibration (NPT) and MD trajectories were optimized using LanM EF1 peptide with Ca^2+^ (Figure S10). Two nanoseconds of NPT and 5 ns of MD sampling were used as they give reasonably converged binding energies. The van der Waals contribution to the chelator binding energies is relatively small and does not vary significantly; for Ca^2+^, Δ*G*_VdW_ = 9.5 kJ mol^–1^ in EDTA, 9.2 kJ mol^–1^ in NTA, 9.1 kJ mol^–1^ in EGTA and 9.1 kJ mol^–1^ in LanM EF1. Consequently, we chose to use a fixed −Δ*G*_VdW_ contribution of 9 kJ mol^–1^ for all metal ions in both the LanM EF1 and CaM EF1 systems to reduce computational cost. The Δ*G*_ele+pol_ portion of the TI calculations was run in triplicate for each system to improve sampling and allow error estimation. The van der Waals contribution (−Δ*G*_VdW_) to Δ*G*_b_^sim^ was not included in the error analysis. The average binding energies for LanM EF1 and CaM EF1 are shown in Table 3.

**Table 3:** Mean Binding Energies (kJ mol^–1^) for Metal Binding to the LanM and CaM EF1 Loop Peptides

metal	exp^14,31−33^	12-6LJ CM/IOD^a^	12-6-4LJ	12-6-4LJ Ch-BE
LanM				
Ca^2+^	–18.0	–67.4	–52.3	–55.5 ± 2.7
Mg^2+^	NA^b^	–119.2	–84.3	–39.9 ± 8.6
Y^3+^	–61.4	–205.6	–151.7	–73.2 ± 14.1
La^3+^	–64.3	–159.6	–127.9	–95.8 ± 7.8
CaM				
Ca^2+^	–33.9	–53.6	–71.5	–54.1 ± 1.9
Mg^2+^	–22.8	–121.1	–100.3	–57.8 ± 3.2
Y^3+^	NA^b^	–189.5	–148.3	–74.7 ± 7.5
La^3+^	–45.3	–152.3	–126.7	–89.1 ± 3.2

a 12-6LJ CM was used for divalent and 12-6LJ IOD for trivalent ions.

b Not available.

As seen in Table 3, all simulations significantly overestimate the binding energies. As the experimental values are the average affinity for the 3–4 EF loop binding sites in LanM and CaM, some of this error may reflect differences in affinity between the different EF loops in each protein. The 12-6LJ CM/IOD parameter sets performed the worst in each case, except for Ca^2+^ binding to CaM EF1. The 12-6-4LJ Ch-BE parameters performed significantly better than the default 12-6-4LJ parameters, but still significantly overestimate the binding energy for all cases. The average errors in binding energy are 84.6, 63.1 and 29.0 kJ mol^–1^ for 12-6LJ CM/IOD, 12-6-4LJ, and 12-6-4LJ Ch-BE, respectively, and there is a 2.9-fold and 2.2-fold increase in accuracy for the 12-6-4LJ Ch-BE parameter set compared to 12-6LJ CM/IOD and 12-6-4LJ, respectively. The observed standard deviations in Δ*G*_b_^sim^ values (between triplicate Δ*G*_ele+pol_ simulations) are relatively low, rarely exceeding 9 kJ mol^–1^, indicating that these simulations give consistent results when starting from the same input geometry.

The 12-6-4LJ Ch-BE parameters were the only parameters that reproduced the experimentally observed order of binding affinities of LanM EF1 with three metal ions: La^3+^ > Y^3+^ > Ca^2+^.^14^ Although there are no reported LanM EF-Mg^2+^ binding energies, the data in Table 3 predict that the affinity is weaker than that for Ca^2+^. This is usually the case for CaM EF-hands.^31,32^ For CaM EF1, all three parameter sets failed to predict the correct order of affinities for the three metal ions: La^3+^ > Ca^2+^ > Mg^2+^ (Table 3). Instead, they predict the same binding affinity order of: La^3+^ > Mg^2+^ > Ca^2+^. Nevertheless, the 12-6-4LJ Ch-BE parameters performed significantly better than the other parameter sets. There is no experimental data for Y^3+^ binding to CaM, but the predicted binding energy is 14.4 kJ mol^–1^ weaker than that for La^3+^, suggesting CaM EF1 has a binding preference for La^3+^.

To gain better insight into EF-hand metal ion coordination, the IODs and CNs were calculated using the same three parameter sets and these values are given in Table 4. Additionally, to compare the metal ion coordination of isolated EF-hands to the EF-hand motifs in a full-length protein and to further test the 12-6-4LJ Ch-BE parameters, 10 ns MD simulations on full-length LanM were performed to determine the IODs and CNs for each of the EF1, EF2 and EF3 binding sites with bound Ca^2+^, Mg^2+^, Y^3+^, and La^3+^ (Tables 4 and S8). In general, the new 12-6-4LJ Ch-BE parameters produced very similar results to the existing 12-6-4LJ parameters.

**Table 4:** Ion-Oxygen Distances (IODs in Å) and Coordination Numbers (CNs) of Metal Ions in Full-Length LanM and the EF1 Loop Peptides of LanM and CaM

	12-6LJ CM/IOD^a^		12-6-4LJ		12–6-4LJ Ch-BE^b^	
metal	CN	IOD	CN	IOD	CN	IOD
LanM EF1 peptide						
Ca^2+^	8.3–8.7^c^	2.43	8.1	2.39	8.0–8.5^c^	2.40
Mg^2+^	6.0	1.94	7.0	2.08	6.0–6.7^c^	2.07
Y^3+^	9.0	2.28	9.0	2.30	9.0–9.3^c^	2.31
La^3+^	10.0	2.45	10.0	2.50	10.0	2.49
LanM EF1 in full-length LanM						
Ca^2+^	7.7–7.8^c^	2.41	8.0–8.1^c^	2.39	7.8–8.0^c^	2.38
Mg^2+^	6.0	1.94	6.5–6.8^c^	2.07	6.1–6.3^c^	2.06
Y^3+^	9.0	2.29	9.0	2.29	9.0	2.29
La^3+^	9.9	2.44	10.0	2.46	10.0	2.48
CaM EF1 peptide						
Ca^2+^	8.2	2.42	8.0	2.40	7.7–8.0^c^	2.40
Mg^2+^	6.0	1.94	7.0	2.09	6.0	2.05
Y^3+^	9.0	2.29	9.1	2.31	9.0	2.30
La^3+^	10.0	2.45	10.0	2.48	10.0	2.49

a 12-6LJ CM for divalent and 12-6LJ IOD for trivalent ions.

b IODs were calculated as the average from triplicate simulations, whereas for CNs, if different values were obtained between simulations, the full observed range is shown.

c No clear minima were observed so the range is given.

As seen in Table 4, Ca^2+^ and Mg^2+^ ion coordination in LanM and CaM EF-hands is not represented by a single binding mode as the CN values are noninteger. Y^3+^ and La^3+^ ions displayed consistent CN numbers in both LanM EF1 and CaM EF1 being ∼9 and ∼10, respectively. Inspection of the simulations revealed that some coordinating residues were fluctuating between monodentate and bidentate ligating modes (Figure S11). This is likely to be physically realistic (e.g., as is observed in CaM, PDB 1CFF). However, if a fixed integer CN is required while retaining the ability to switch ligands this could be achieved by applying the cationic dummy atom model (CDAM) in combination with re-parameterization of the ligating atom *C_ij_* terms. The CDAM partitions the metal ion charge between itself and dummy atoms surrounding the central metal ion in a predefined geometry, which interacts with the surrounding atoms and can freely exchange ligands.^37^ Recently, CDAM was combined with 12-6-4LJ potential for Mg^2+^, Fe^3+^, Al^3+^, and Cr^3+^ ions in a predefined octahedral geometry and was shown to reproduce HFE, IOD, and CN values in a water solution.^38^ Alternatively, it has recently been suggested that modified angle and torsion parameters on ligands combined with the double decoupling method^39^ might improve the TI simulation performance for transition metal binding to coordination complexes and proteins.^40^ This approach should be applicable to the study of metal binding to CaM and LanM and in future work it would be interesting to see if this improves the computed binding energies and/or CN values.

The computed IODs from the Ca^2+^, Mg^2+^, Y^3+^, and La^3+^ simulations with the EF1 peptides and full-length LaM do not significantly differ between the 12-6-4LJ and 12-6-4LJ Ch-BE parameters. These IODs varied within ±0.02 Å in cases where CN remained the same (Table 4). The 12-6LJ CM/IOD parameter set usually gave rise to lower IODs for Mg^2+^, Y^3+^, and La^3+^, while the Ca^2+^ IODs were relatively similar to those computed with the 12-6-4LJ forcefield. All three parameter sets produced relatively accurate average IODs for Y^3+^ in LanM EF1 (∼2.3 Å) compared to experimental structures in PDB 6MI5 (2.2–2.4 Å depending on the ligating group).^15^ The Ca^2+^ IODs were also consistent with experimental CaM structures. Experimental IODs of 2.42 Å for monodentate and 2.41 Å for bidentate ligands in EF1 of PDB 1CLL are in good agreement with the 12-6-4LJ Ch-BE average IODs of 2.40 Å for two bidentate and four monodentate ligands.^16^ For Mg^2+^, the 12-6-4LJ Ch-BE average IOD of 2.05 Å is comparable with the experimental monodentate ligation distance of 2.11 Å in EF1 of PDB 3UCW.^41^ No crystal structures of LanM or CaM with La^3+^ are available for comparison.

## Conclusions

We have demonstrated a method of generating *C_ij_* parameters for 12-6-4LJ MM forcefields that allows parametrization of specific ligating groups in order to tune binding energies computed by TI. The new *C_ij_* coefficients were tested on a series of chelators: EDTA, NTA, EGTA, and EF1 loop peptides from LanM and CaM proteins and showed significant improvements in computed binding energies relative to existing forcefields. The new parameters also produce CN and IOD values that are in good agreement with experimental values. The parametrization method should be extensible to a range of other systems and could be readily adapted to tune properties other than binding energies.

## Supporting Information Available

Additional methodological information, reference data, and analysis (PDF)
