# Supplementary material for: Chelator-Based Parameterization of the 12-6-4 Lennard-Jones Molecular Mechanics Potential for More Realistic Metal Ion–Protein Interactions
Source: J Chem Theory Comput. 2022 Mar 23;18(4):2367–74. doi: 10.1021/acs.jctc.1c00898 (PMC9171819; doi:10.1021/acs.jctc.1c00898)
Supplement: Supplementary file 1 — ct1c00898_si_001.pdf [file ct1c00898_si_001.pdf]

Supporting information

Chelator-based parameterization of the 12-6-4LJ molecular mechanics potential for more realistic metal ion-protein interactions

*Paulius Kantakevičius, Calvin Mathiah, Linus O. Johannissen and Sam Hay**

Manchester Institute of Biotechnology and Department of Chemistry, The University of Manchester, Manchester, United Kingdom. sam.hay@manchester.ac.uk.

Additional details: Molecular dynamics simulations

For chelators: EDTA, NTA and EGTA the GAFF atom types (Version 1.81) were generated using the Antechamber package from AmberTools19,[1-3] with charges calculated using the AM1-BCC charge model.[4] The obtained charges of all atom types are displayed in Table S2. The NPT and MD sampling lengths were optimized using EDTA and La^{3+} ion as a model system (Figure S9) suggesting an optimal NPT length of 0.5 ns and sampling length of 1.5 ns.

For full LanM MD simulations that were used in CN and IOD calculations State 1 from NMR structure (PDB 6MI5) with one metal ion in each: EF1, EF2 and EF3 hand was used as initial structure.[5] EF4 was left without a metal ion as it contains the lowest affinity towards metal ions and it did not contain a metal ion in the original structure. To produce full LanM MD trajectory for IOD and CN calculations three step EM was done with 2000/3000, 2000/3000, 20000/30000 steepest and conjugate descent, respectively. NVT equilibration of 1 ns was done followed by 2 ns NPT equilibration and 10 ns unconstrained MD simulations.

From Figure S10 it was observed that 2 ns of NPT and 5 ns of sampling gives reasonably converged binding energies. Sampling lengths above 5 ns did not improve the results as the plateau was already reached, whereas NPT longer than 2 ns gives a small ~2 kJ mol^{-1} improvement when the NPT length is extended to 5 ns. However, this improvement is not large enough to justify the increase in processing time as the majority of convergence (~10 kJ mol^{-1}) happened during the first 2 ns of NPT. The same length NPT and MD sampling was applied to CaM EF1 as the geometry and amino acid sequence (and thus the system size) is highly similar to that of Lan EF1 as seen in Figures 1 and S1, respectively

Alternative equations for different coordinating atom ratios

The coordination numbers and coordinating atom ratios in Eq. 7 may differ between metal ions. For example, it was observed that EDTA can have different coordination states with Ca^{2+} ions.[6] During parametrization one can change the ligating atom ratios to account for this behavior. For example, if EDTA uses 3 oxygen atoms and 2 nitrogen atoms for Ca^{2+} ligation (and NTA uses the maximum 3 oxygen atoms and 2 nitrogen atoms), one would modify Eq. 7 to:

$$\begin{cases} \Delta G_{\mathrm{b}}^{sim'}(EDTA, C_{ij} = 0{,}0) + m(\mathrm{EDTA,O})C_{ij}(\mathrm{O}) + m(\mathrm{EDTA,N})C_{ij}(\mathrm{N}) = \Delta G_{\mathrm{b}}^{exp}(\mathrm{EDTA}) \\ \Delta G_{\mathrm{b}}^{sim'}(\mathrm{NTA}, C_{ij} = 0{,}0) + m(\mathrm{EDTA,O})C_{ij}(\mathrm{O}) + 1/2\, m(\mathrm{EDTA,N})C_{ij}(\mathrm{N}) = \Delta G_{\mathrm{b}}^{exp}(\mathrm{NTA}) \end{cases}$$

This in turn would produce C_{ij}(O) = 14.5 and C_{ij}(N) = 160.2 (cf. 29.2 and 110.6 in Table 2). It is also possible to assume non-integer metal ion coordination and changing ratios accordingly (i.e., assuming that EDTA on average uses 3.7 oxygen atoms for coordination, one would change the ratio to 3/3.7), giving C_{ij}(O) = 23.3 and C_{ij}(N) = 130.6:

$$\begin{cases} \Delta G_{\mathrm{b}}^{sim'}\left(EDTA, C_{ij} = 0{,}0\right) + m(\mathrm{EDTA,O})C_{ij}(\mathrm{O}) + \ m(\mathrm{EDTA,N})C_{ij}(\mathrm{N}) = \Delta G_{\mathrm{b}}^{exp}(\mathrm{EDTA}) \\ \Delta G_{\mathrm{b}}^{sim'}\left(\mathrm{NTA}, C_{ij} = 0{,}0\right) + \dfrac{3}{3.7} m(\mathrm{EDTA,O})C_{ij}(\mathrm{O}) + 1/2\ m(\mathrm{EDTA,N})C_{ij}(\mathrm{N}) = \Delta G_{\mathrm{b}}^{exp}(\mathrm{NTA}) \end{cases}$$

Y^{3+} binding to EGTA

Visual inspection of the relevant MD trajectories indicated that with both default and new C_{ij} coefficients Y^{3+} ions were coordinated inconsistently by EGTA. In some simulations EGTA would use most of its ligating groups and one water molecule for coordination of metal ion, whereas in other cases the Y^{3+} ion would move outwards and become coordinated by a higher number of water molecules as shown in Figure S8. Interestingly, EGTA systems with more water molecules coordinating the Y^{3+} ion were more energetically favorable as TI calculated higher binding energies. The energy difference between these states varied by over 50 kJ mol^{-1} between replicate runs with new 12-6-4LJ C_{ij} coefficients producing a lowest binding energy of -42.1 kJ mol^{-1} and highest of -95.6 kJ mol^{-1}. Similar variation was observed between replicate runs with default 12-6-4LJ parameter set providing the lowest binding energy of -91.8 kJ mol^{-1} ("normal" state) and the highest of -150.3 kJ mol^{-1} ("EGTA-solvent hydrogen bonded" state). These energetic differences may be explained by a water molecule forming hydrogen bonds to the ether oxygen and carboxylate group of EGTA and another water molecule stabilizing metal ion coordinating carboxylate groups, which likely stabilized the "EGTA-solvent hydrogen bonded" geometry thus increasing the binding energy (Figure S8). To further investigate this, EGTA-Ca^{2+} simulations were inspected and no such ligand variation was observed as in all cases Ca^{2+} ions used ligands provided by EGTA as observed in the crystal structure.[7]

Table S1. Experimental metal ion hydration free energies (HFE) and binding energies (in kJ mol^{-1}) for EDTA, NTA, EGTA, LanM and CaM. Experimental logK_1 values for EDTA, NTA and EGTA and K_d values (in M) for LanM and CaM are included in (parenthesis).

Metal	Ion HFE[8]	Binding energy EDTA[9]	NTA[10]	EGTA[11]	LanM EF1[12]	CaM EF1[13-15]
Ca^{2+}	-1505	-60.8, (10.65)	-37.5, (6.57)	-62.8, (11.00)	-18.0, (7.1 x 10^{-4})	-39.9, (1 x 10^{-7})
Mg^{2+}	-1830	-50.1, (8.79)	-30.6, (5.36)	-29.7, (5.21)	NA [a]	-22.8, (1 x 10^{-4})
Y^{3+}	-3450	-103.2, (18.08)	-65.5, (11.48)	-96.0, (16.82)	-61.4, (1.7 x 10^{-11})	NA [a]
La^{3+}	-3145	-87.6, (15.36)	-59.1, (10.36)	-90.1, (15.79)	-64.3, (5.3 x 10^{-12})	-45.3, (1.17 x 10^{-8})

[a] Not available.

Table S2. Atom types and charges generated by Antechamber for chelators EDTA, NTA and EGTA. Atom labels are shown in Figure S3.

Atom label in Figure S3	GAFF atom type	Charge		
		EDTA	NTA	EGTA
O	o	-0.8922	-0.9003	-0.8797
C	c	0.9123	0.8996	0.8976
C3	c3	0.0711	0.0758	0.0648
H1	h1	0.0186	0.0282	0.0282
N3	n3	-0.6946	-0.6926	-0.7106
C3a	c3	0.1493		0.1668
H1a	h1	0.0367		0.0552
C3b	c3			0.1254
H1b	h1			0.0189
C3c	c3			0.1019
H1c	h1			0.041
Os	os			-0.4311

Table S3. Summary of energy minimization (EM), NVT, NPT and molecular dynamics (MD) sampling lengths per each λ step used for thermodynamic integration in different systems.

System	λ steps (Ele+pol, VdW)	EM steepest, conjugate descent (number of cycles)	NVT (ps)	NPT (ps)	MD sampling (ps)
Water box	9, 9	500, 500 (1000) [b]	20	100	900
EDTA, NTA, EGTA	12 (19), 9 [a]	500, 500 (1000) [b]	20-50 [c]	100-500 [c]	900-1900 [c]
EF-hand	12, 9	2000, 3000 (5000) [b]	500	2000	5000

[a] in several cases 19 λ steps with 0.05 step size were used with rectangular integration, however 12 λ steps with Gaussian integration is more computationally efficient; [b] for VdW removal steepest descent cannot be used, only conjugate descent was used with the number of steps indicated in parentheses; [c] there was some variation in NVT, NPT and MD sampling lengths between simulations in chelators – ranges are given in the table. The optimal NPT length being 0.5 ns and MD length 1.5 ns as shown in Figure S9.

Table S4. Coordination number (CN) and ion-oxygen distance (IOD in Å) values for metal ions in water solution calculated from λ=0.00922 thermodynamic integration (TI) simulations.

Metal	exp[16,17]		12-6LJ CM/IOD [a, b]		12-6LJ HFE [b]		12-6-4LJ [b]	
	CN	IOD	CN	IOD	CN	IOD	CN	IOD
Water box								
Ca^{2+}	8	2.46	8/8 [c]	2.50/ 2.49 [c]	7.3-7.4/ 7.4 [c]	2.33/ 2.33 [c]	8/8 [c]	2.47/ 2.46 [c]
Mg^{2+}	6	2.09	6/6 [c]	2.04/ 2.03 [c]	6/6 [c]	1.95/ 1.95 [c]	6/6 [c]	2.09/ 2.09 [c]
Y^{3+}	8	2.36	8.9-9	2.36	~8-9	2.16	9/9 [c]	2.36/ 2.36 [c]
La^{3+}	8-9.1	2.52	9	2.49	9	2.40	9.4-9.6/ 9.7 [c]	2.52/ 2.53 [c]

[a] 12-6LJ CM parameter set was used for divalent ions and 12-6LJ IOD for trivalent ions; [b] IODs were calculated as an average of 3 runs whereas for CNs if different values were obtained between runs the full observed range is shown; [c] first number for CN and IOD for Ca^{2+} and Mg^{2+} ions are calculated from the λ=0.00922 TI simulations, while the second number are taken from the parameterization studies. Trivalent ions did not contain values in parameterization studies for 12-6LJ IOD and HFE parameter sets.[18-20]

Table S5. Hydration free energies (HFE, in kJ mol^{-1}) calculated using thermodynamic integration with three different metal parameter sets.

Metal	exp[8]	12-6LJ CM/IOD [a]	12-6LJ HFE	12-6-4LJ
HFE				
Ca^{2+}	-1505	-1402.0 ± 0.9	-1510.0 ± 0.1	-1507.5 ± 0.5
Mg^{2+}	-1830	-1727.0 ± 1.2	-1833.0 ± 0.6	-1829.1 ± 0.3
Y^{3+}	-3450	-3180.9 ± 1.7	-3416.2 ± 0.2	-3451.2 ± 0.9
La^{3+}	-3145	-3007.0 ± 2.7	-3143.7 ± 2.9	-3137.4 ± 1.5

[a] 12-6LJ CM parameter set for divalent ions and 12-6LJ IOD for trivalent ions.

Table S6. Ethylenediaminetetraacetic acid (EDTA) metal ion binding energies (kJ mol^{-1}) calculated using thermodynamic integration with four parameter sets.

Metal	exp[9]	12-6LJ CM/IOD [a]	12-6LJ HFE	12-6-4LJ	12-6-4LJ Ch-BE [b]
Ca^{2+}	-60.8	-55.4 ± 5.2	-89.2 ± 7.9	-56.0 ± 1.3	-66.2 ± 5.6
Mg^{2+}	-50.1	-135.0 ± 10.5	-173.8 ± 4.6	-88.1 ± 0.6	-54.4 ± 5.1
Y^{3+}	-103.2	-215.4 ± 12.4	-343.2 ± 3.9	-156.0 ± 12.7	-107.5 ± 2.3
La^{3+}	-87.6	-160.7 ± 6.2	-201.9 ± 8.0	-123.3 ± 4.3	-96.2 ± 10.3

[a] 12-6LJ CM used for divalent ions and 12-6LJ IOD for trivalent ions; [b] 12-6-4LJ Ch-BE is the parameter set developed in this study.

Table S7. Absolute nitriloacetic acid (NTA) and egtazic acid (EGTA) binding energies with default and new C_{ij} coefficients of 12-6-4LJ potential in kJ mol^{-1}.

Metal	exp[10,11]	12-6-4LJ	12-6-4LJ Ch-BE
NTA			
Ca^{2+}	-37.5	-32.7 ± 0.7	-31.4 ± 3.0
Mg^{2+}	-30.6	-84.9 ± 5.9	-49.5 ± 9.5
Y^{3+}	-65.5	-148.9 ± 1.9	-113.8 ± 4.8
La^{3+}	-59.1	-115.4 ± 6.3	-94.1 ± 4.3
EGTA			
Ca^{2+}	-62.8	-54.2 ± 8.5	-66.7 ± 7.1
Mg^{2+}	-29.7	-85.1 ± 12.4	-56.1 ± 15.1
Y^{3+}	-96.0	-125.0 ± 30.0	-95.6 ± 28.4
La^{3+}	-90.1	-120.7 ± 11.4	-73.3 ± 2.1

Table S8. Ion-oxygen distances (IODs in Å) and coordination numbers (CNs) of metal ions in EF2 and EF3 loops of full-length LanM.

	12-6LJ CM/IOD [a]		12-6-4LJ		12-6-4LJ Ch-BE	
Metal	CN	IOD	CN	IOD	CN	IOD
EF2						
Ca^{2+}	8.0	2.41	8.3-8.4 [b]	2.39	7.8-8.0 [b]	2.39
Mg^{2+}	6.0	1.95	7.0	2.09	6.0	2.06
Y^{3+}	8.8-8.9 [b]	2.26	9.0	2.28	9.0	2.28-
La^{3+}	9.8	2.43	10.0	2.47	10.0	2.47
EF3						
Ca^{2+}	8.0	2.41	8.0	2.38	8.0	2.38
Mg^{2+}	6.0	1.95	6.9	2.08	6.0	2.05
Y^{3+}	9.0	2.27	9.0	2.28	9.0	2.29
La^{3+}	10.0	2.45	10.0	2.46	10.0	2.49

[a] 12-6LJ CM for divalent and 12-6LJ IOD for trivalent ions; [b] no clear minima were observed so the range is given.

Calmodulin		Lanmodulin	
EF1 20	DKDGDGTI TTKE 31	EF1 35	DPDKDGTI DLKE 46
EF2 56	DADGNGTI DFPE 67	EF2 59	DPDKDGTLDAKE 70
EF3 93	DKDGNGYI SAAE 104	EF3 84	DPDNDGTLDKKE 95
EF4 129	DI DGDGQVNYEE 140	EF4 108	NPDNDGTI DARE 119

Figure S1. Calmodulin (PDB 1CLL) and lanmodulin (PDB 6MI5) EF-hand motif sequence comparison.[5,21]

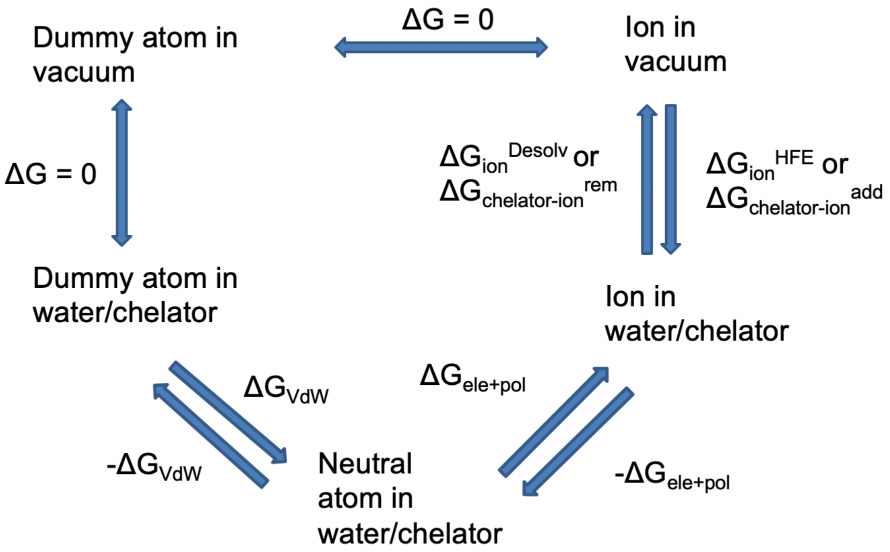

$$\Delta G_{ion}{}^{HFE}\left(or\ \Delta G_{chelator-ion}{}^{add}\right) = \Delta G_{ele+pol} + \Delta G_{VdW}$$
$$\Delta G_b = \Delta G_{chelator-ion}{}^{add} - \Delta G_{ion}{}^{HFE} = -(\Delta G_{chelator-ion}{}^{rem} - \Delta G_{ion}{}^{Desolv})$$
$$\Delta G = G(\lambda = 1) - G(\lambda = 0) = \int_0^1 \langle\frac{\delta V}{\delta\lambda}\rangle_i\, d\lambda \ = \sum w_i \langle \delta V/\delta\lambda\rangle_i$$

Figure S2. Thermodynamic cycle for ion hydration free energy and chelator-ion binding energy calculations.

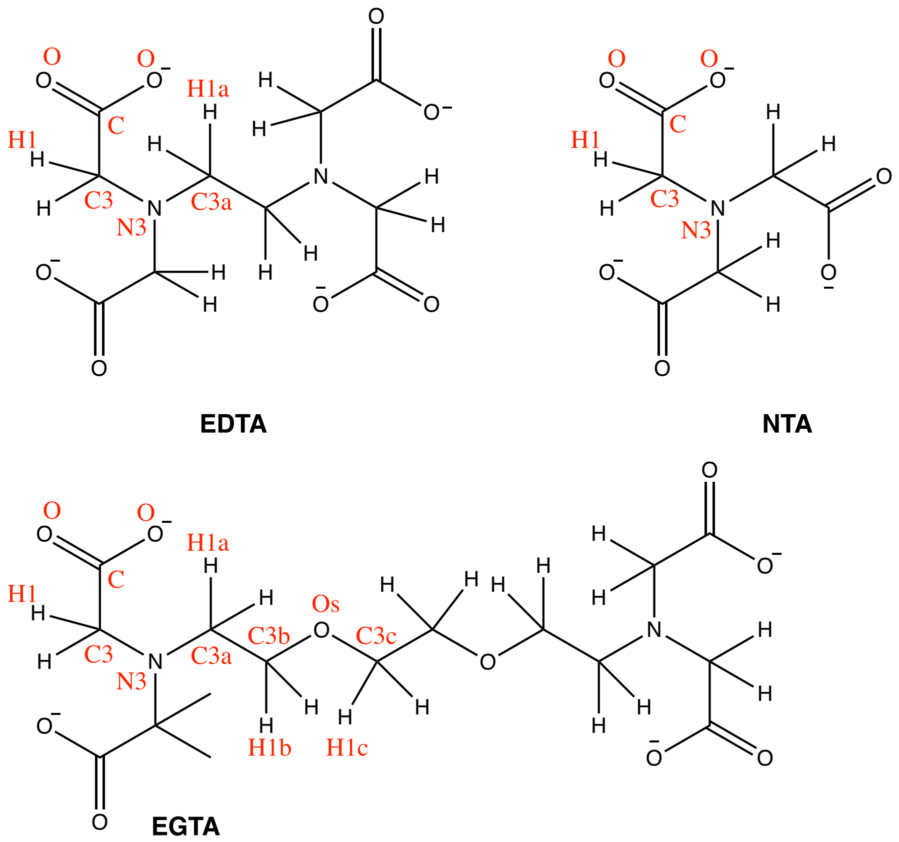

Figure S3. Atom labels used in Table S2.

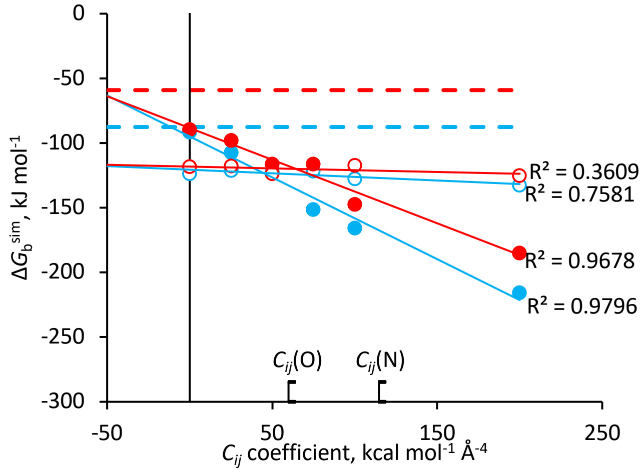

Figure S4. Computed binding energies for the chelation of La^{3+} by EDTA (blue) and NTA (red) as a function of the ligating oxygen and nitrogen 12-6-4LJ C_{ij} values. Filled symbols are for oxygen and open symbols for nitrogen. Solid lines are linear fits to the data and the horizontal dashed lines are the experimental ΔG_b^{exp} values from Table 1. The default C_{ij} values are labeled.

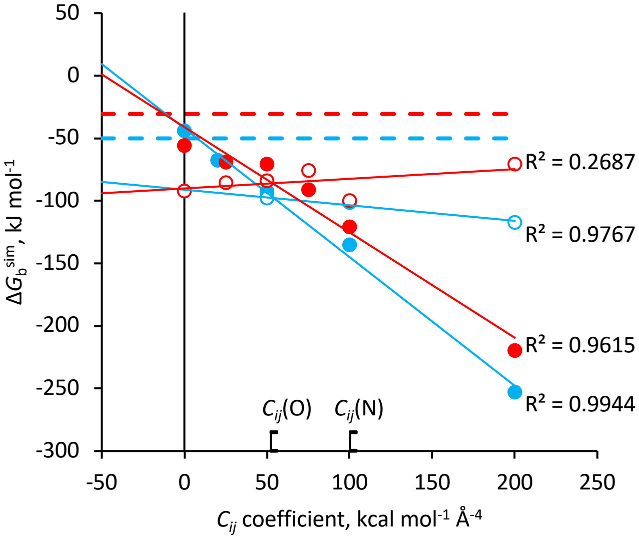

Figure S5. Computed binding energies for the chelation of Mg^{2+} by EDTA (blue) and NTA (red) as a function of the ligating oxygen and nitrogen C_{ij} values and the 12-6-4LJ force field. Filled symbols are for oxygen and open symbols for nitrogen. Solid lines are linear fits to the data and the horizontal dashed lines are the experimental values from Table 1. The default C_{ij} values are labeled.

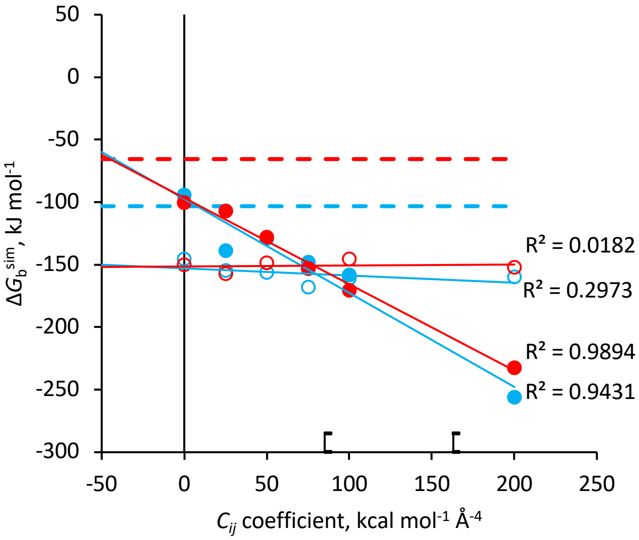

Figure S6. Computed binding energies for the chelation of Y^{3+} by EDTA (blue) and NTA (red) as a function of the ligating oxygen and nitrogen C_{ij} values and the 12-6-4LJ force field. Filled symbols are for oxygen and open symbols for nitrogen. Solid lines are linear fits to the data and the horizontal dashed lines are the experimental values from Table 1. The default C_{ij} values are labeled.

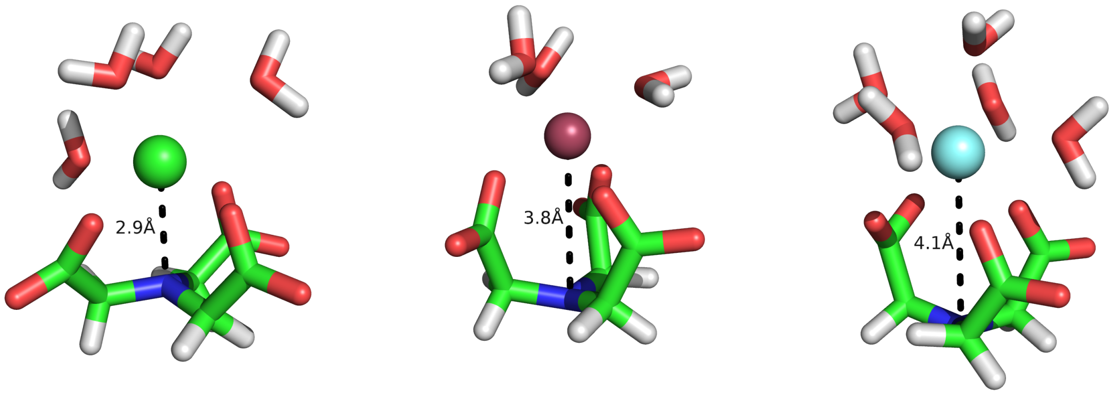

Figure S7. NTA coordination of metal ions. Snapshots were taken from 12-6-4LJ with default C_{ij} coefficients λ=0.00922 thermodynamic integration simulations where 99% of the charge are present. Metal ions: Ca^{2+} (green), Mg^{2+} (raspberry) and Y^{3+} (aqua).

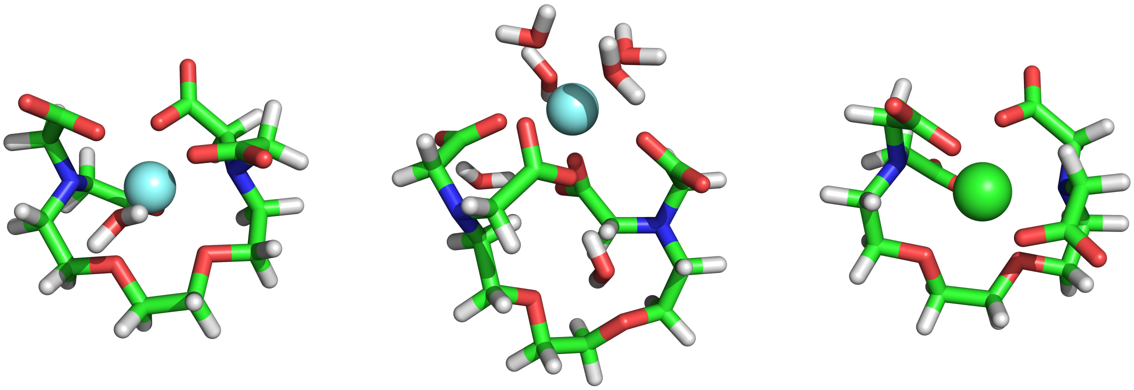

Figure S8. Different EGTA metal ion ligating geometries. Snapshots were taken from 12-6-4LJ with default C_{ij} coefficients λ=0.00922 simulations where 99% of the charge are present. Y^{3+} displayed varied coordination modes – "normal" (left) and "EGTA-solvent hydrogen bonded" (middle), which differed in coordinating water molecule and EGTA ligating group number. Metal ions like Ca^{2+} showed one major coordination state – "normal" (right), which only used ligating groups provided by EGTA.

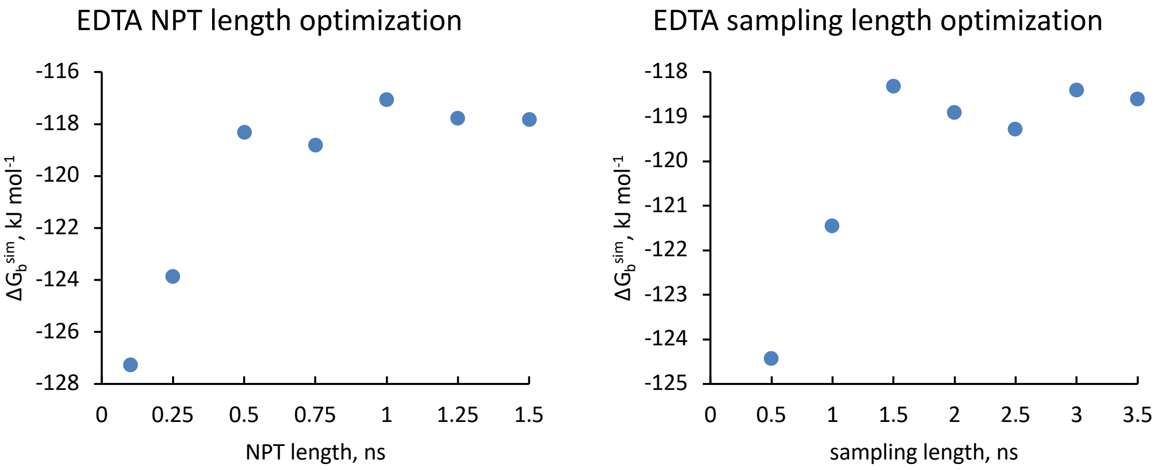

Figure S9. EDTA and metal ion NPT and sampling length optimization. EDTA with La^{3+} ion was used as a model system to determine NPT and sampling lengths per λ step of TI required to produce consistent binding energies. If NPT was varied then sampling length was fixed at 1.5 ns, while if sampling length was varied then NPT was fixed at 0.5 ns.

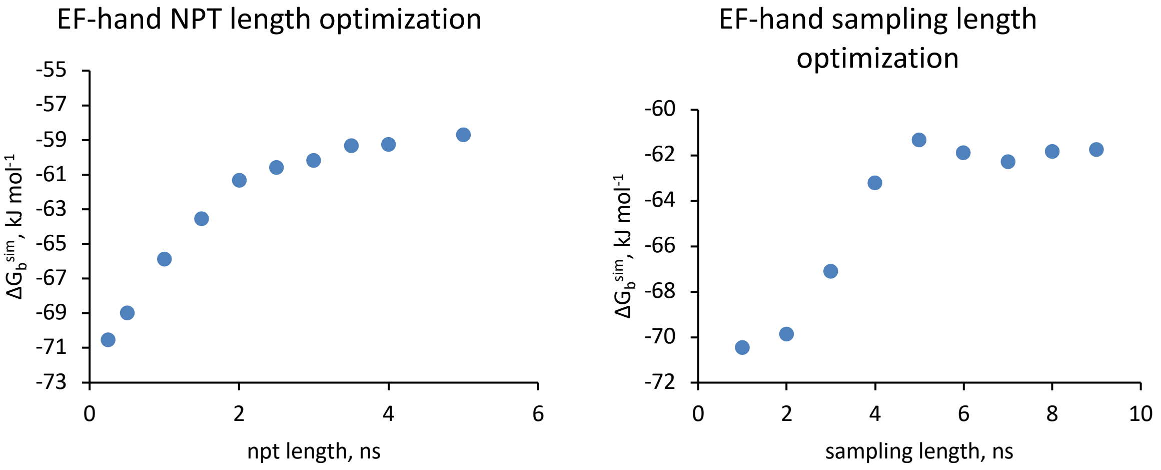

Figure S10. EF-hand and metal ion NPT and sampling length optimization. Lanmodulin EF-hand 1 with Ca^{2+} ion was used as a test system to determine NPT and sampling lengths per λ step of TI required to produce consistent binding energies. If NPT was varied then sampling length was fixed at 5 ns, while if sampling length was optimized then NPT was fixed at 2 ns.

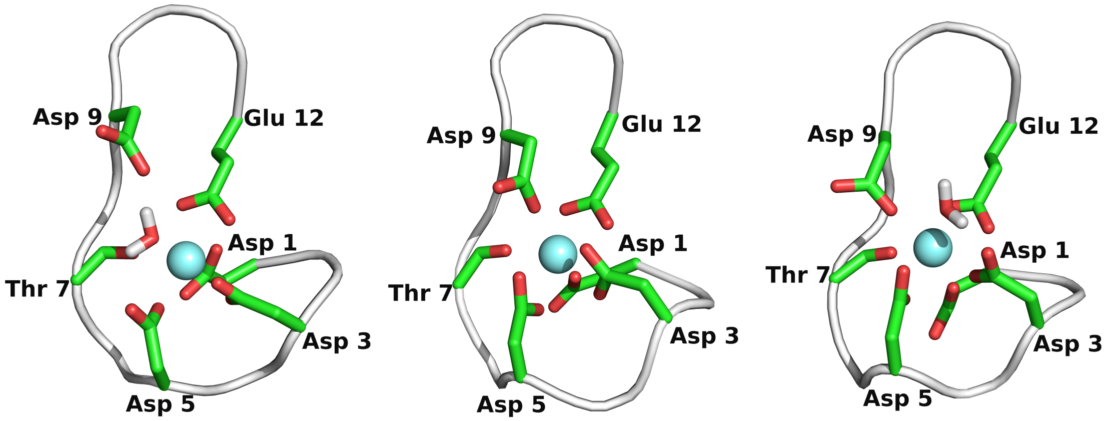

Figure S11. Comparison of computed lanmodulin EF-hand 1 Y^{3+} (LanM EF1) coordination frameworks. 12-6LJ IOD (left), 12-6-4LJ (middle) and 12-6-4LJ Ch-BE (right) representative structures were taken from MD trajectories of full-length LanM with Y^{3+} ions. The peptide backbones are shown as ribbons and the coordinating residues ligating groups as sticks. The 12-6-4LJ default and 12-6-4LJ Ch-BE parameters produced ligation frameworks in which Asp 9 directly coordinated the Y^{3+} ion, while in the 12-6LJ IOD simulation Asp 9 hydrogen bonded to a water molecule that coordinated Y^{3+} instead. Both of these Asp 9 coordination modes were seen in the experimental structures of LanM EF1 as in 3 out of 12 states Asp 9 was used for direct Y^{3+} coordination and in 9 out of 12 states Asp 9 was not used in direct coordination.[5] Interestingly, in 12-6-4LJ Ch-BE simulation Asp 3 instead of Asp 9 hydrogen bonded to the water molecule that coordinated Y^{3+}, which was not observed in the experimental states.

Additional References

1. Case, D. A.; Ben-Shalom, I. Y.; Brozell, S. R.; Cerutti, D. S.; Cheatham, T. E.; III, Cruzeiro, V. W. D.; Darden, T. A.; Duke, R. E.; Ghoreishi, D.; Giambasu, G.; Giese, T.; Gilson, M. K.; Gohlke, H.; Goetz, A. W.; Greene, D.; Harris, R.; Homeyer, N.; Huang, Y.; Izadi, S.; Kovalenko, A.; Krasny, R.; Kurtzman, T.; Lee, T. S.; LeGrand, S.; Li, P.; Lin, C.; Liu, J.; Luchko, T.; Luo, R.; Man, V.; Mermelstein, D. J.; Merz, K. M.; Miao, Y.; Monard, G.; Nguyen, C.; Nguyen, H.; Onufriev, A.; Pan, F.; Qi, R.; Roe, D. R.; Roitberg, A.; Sagui, C.; Schott-Verdugo, S.; Shen, J.; Simmerling, C. L.; Smith, J.; Swails, J.; Walker, R. C.; Wang, J.; Wei, H.; Wilson, L.; Wolf, R. M.; Wu, X.; Xiao, L.; Xiong, D. M.; York D. M.; Kollman P. A. AMBER 2019, University of California, San Francisco, **2019**
2. Wang, J.; Wang, W.; Kollman, P. A.; Case, D. A. Automatic atom type and bond type perception in molecular mechanical calculations. *J. Mol. Graphics Modell.* **2006**, *26*, 247–260.
3. Wang, J.; Wolf, R. M.; Caldwell, J. W.; Kollman, P. A.; Case, D. A. Development and testing of a general AMBER force field. *J. Comp. Chem.* **2004**, *25*, 1157– 1174.
4. Jakalian, A., Bush, B.L., Jack, B.D., Bayly, C.I., Fast, Efficient Generation of High-Quality Atomic Charges. AM1-BCC Model: I. Method. *J. Comp. Chem.* **2000**, *21*, 132– 146.
5. Cook, E. C.; Featherston, E. R.; Showalter, S. A.; Cotruvo, J. A. Structural Basis for Rare Earth Element Recognition by Methylobacterium extorquens Lanmodulin. *Biochemistry* **2019**, *58*, 120– 125.

6. Yuan Q., Kong, X. T., Hou, G. L., Jiang, L., Wang, X. B. Electrospray ionization photoelectron spectroscopy of cryogenic [EDTA· M (ii)] 2− complexes (M= Ca, V–Zn): electronic structures and intrinsic redox properties. *Faraday Discuss.* **2019**, 217, 383– 395.
7. Schauer, C. K.; Anderson, O. P. Highly polydentate ligands. 5. Structures of alkaline-earth complexes of the calcium-selective ligand EGTA4- (H4EGTA = 3,12-Bis(carboxymethyl)-6,9-dioxi-3,12-diazatetradecanedioic acid). *Inorg. Chem.* **1988**, *27*, 3118– 3130.
8. Marcus, Y. Thermodynamics of solvation of ions. Part 5.–Gibbs free energy of hydration at 298.15K. *J. Chem. Soc., Faraday Trans.* **1991**, *87*, 2995– 2999.
9. Anderreg, G. Critical Survey of Stability Constants of EDTA Complexes. IUPAC chemical data series, no 14. Pergamon Press, Oxford, U.K. **1977**.
10. Anderegg, G. Critical Survey of Stability-Constants of NTA Complexes. *Pure Appl. Chem.* **1982**, *54*, 2693– 2758.
11. Dojindo Molecular Technologies, Inc. Chelate table of stability constants. https://www.dojindo.co.jp/technical/pdf/Chelate_Table_of_Stability_Constants.pdf (last accessed 02.03.2022)
12. Cotruvo, J. A.; Featherston, E. R.; Mattocks, J. A.; Ho, J. V.; Laremore, T. N. Lanmodulin: A Highly Selective Lanthanide-Binding Protein from a Lanthanide-Utilizing Bacterium. *J. Am. Chem. Soc.* **2018**, *140*, 15056– 15061.
13. Linse, S.; Helmersson, A.; Forsen, S. Calcium-binding to calmodulin and its globular domains. *J. Biol. Chem.* **1991**, *266*, 8050– 8054.
14. Tsai, M. D.; Drakenberg, T.; Thulin, E.; Forsen, S. Is the binding of magnesium (II) to calmodulin significant? An investigation by magnesium-25 nuclear magnetic resonance. *Biochemistry* **1987**, *26*, 3635– 3643.
15. Xu, K.; Yang, X. D.; Wang, K. Metal binding discrimination of the calmodulin Q41C/K75C mutant on Ca2+ and La3+. *Sci. China Chem.* **2010**, *53*, 797– 806.
16. Marcus, Y. Ionic radii in aqueous solutions. *Chem. Rev.* **1988**, *88*, 1475– 1498.
17. Jalilehvand, F.; Spangberg, D.; Lindqvist-Reis, P.; Hermansson, K.; Persson, I.; Sandstrom, M. Hydration of the Calcium Ion. An EXAFS, Large-Angle X-ray Scattering, and Molecular Dynamics Simulation Study. *J. Am. Chem. Soc.* **2001**, *123*, 431– 441.
18. Li, P. F.; Roberts, B. P.; Chakravorty, D. K.; Merz, K. M. Rational Design of Particle Mesh Ewald Compatible Lennard-Jones Parameters for +2 Metal Cations in Explicit Solvent. *J. Chem. Theory Comput.* **2013**, *9*, 2733– 2748.
19. Li, P. F.; Merz, K. M. Taking into Account the Ion-Induced Dipole Interaction in the Nonbonded Model of Ions. *J. Chem. Theory Comput.* **2014**, *10*, 289– 297.
20. Li, P. F.; Song, L. F.; Merz, K. M. Parameterization of Highly Charged Metal Ions Using the 12-6-4 LJ-Type Nonbonded Model in Explicit Water. *J. Phys. Chem. B* **2015**, *119*, 883–895.
21. Chattopadhyaya, R.; Meador, W. E.; Means, A. R.; Quiocho, F. A. Calmodulin structure refined at 1.7 angstrom resolution. *J. Mol. Biol.* **1992**, *228*, 1177– 1192.
